# Supplementary material for: Tetrel Bonds between Phenyltrifluorosilane and Dimethyl Sulfoxide: Influence of Basis Sets, Substitution and Competition
Source: Molecules. 2021 Nov 29;26(23):7231. doi: 10.3390/molecules26237231 (PMC8658981; doi:10.3390/molecules26237231)
Supplement: Supplementary file 1 [file molecules-26-07231-s001.zip › molecules-1474813-supplementary.pdf]

Supplementary Materials

Tetrel Bonds between Phenyltrifluorosilane and Dimethyl Sulfoxide: Influence of Basis Sets, Substitution and Competition

Xiulin An [1], Xin Yang [2] and Qingzhong Li [2,*]

[1] College of Life Science, Yantai University, Yantai 264005, China; anxiulinli@sina.com
[2] The Laboratory of Theoretical and Computational Chemistry, School of Chemistry and Chemical
Engineering, Yantai University, Yantai 264005, China; yangx@ytu.edu.cn
* Correspondence: lqz@ytu.edu.cn

Table S1. Electrostatic energy (E^{ele}), exchange energy (E^{ex}), repulsion energy (E^{rep}), polarization energy (E^{pol}), and dispersion energy (E^{disp}) in the complexes at the MP2/aug-cc-pVDZ level. All are in kJ/mol.

complexes	E^{ele}	E^{ex}	E^{rep}	E^{pol}	E^{disp}
$PhSiF_3\cdots$DMSO	-46.40	-66.59	118.59	-11.62	-9.95
p-NH_2-$PhSiF_3\cdots$DMSO	-45.14	-67.76	119.76	-11.12	-11.70
p-NO_2-$PhSiF_3\cdots$DMSO	-341.30	-367.34	743.08	-206.87	20.44

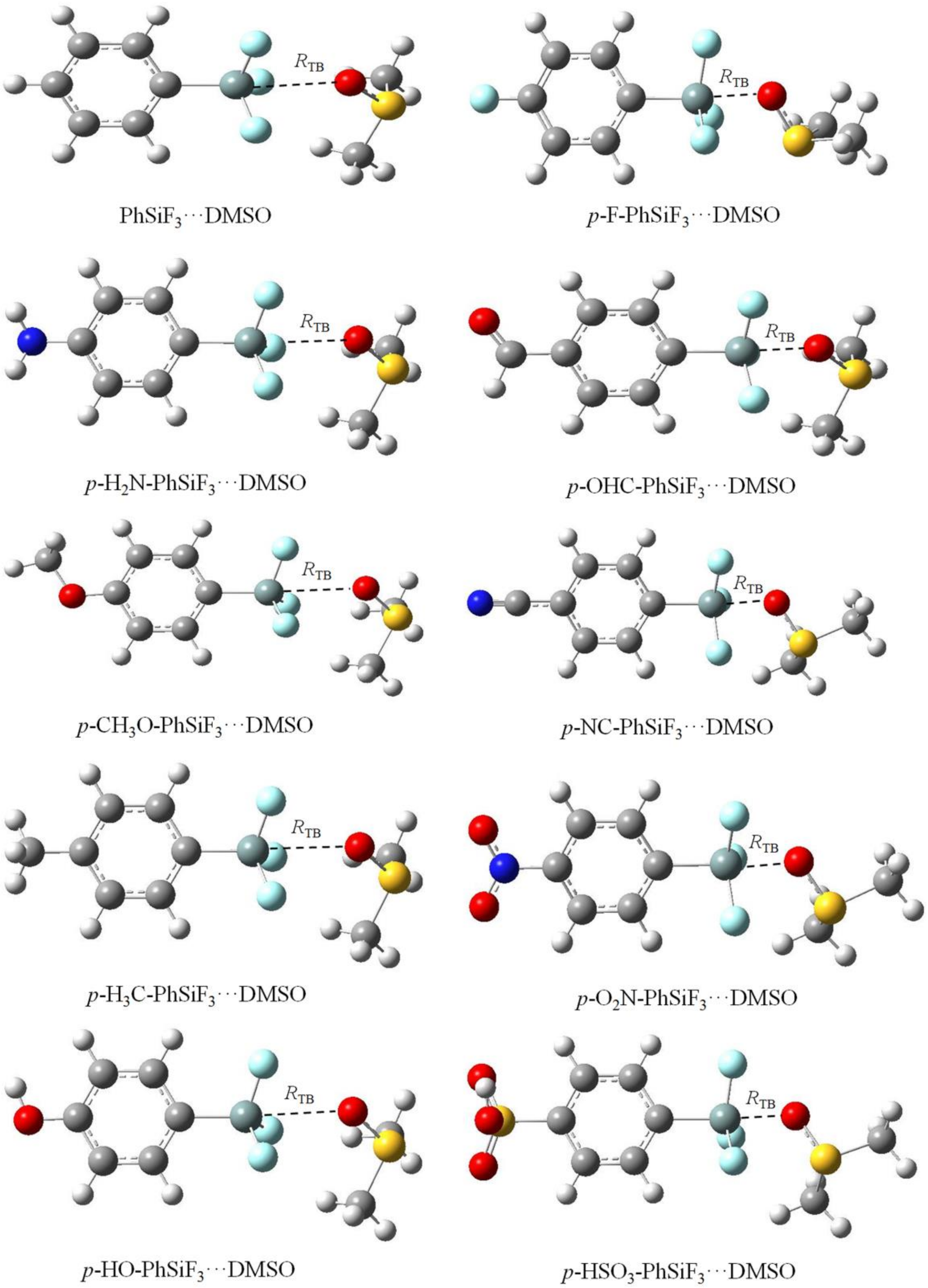

Figure S1 Optimized structures of complexes p-X-PhSiF$_3$···DMSO at the MP2/aug'-cc-pVTZ level

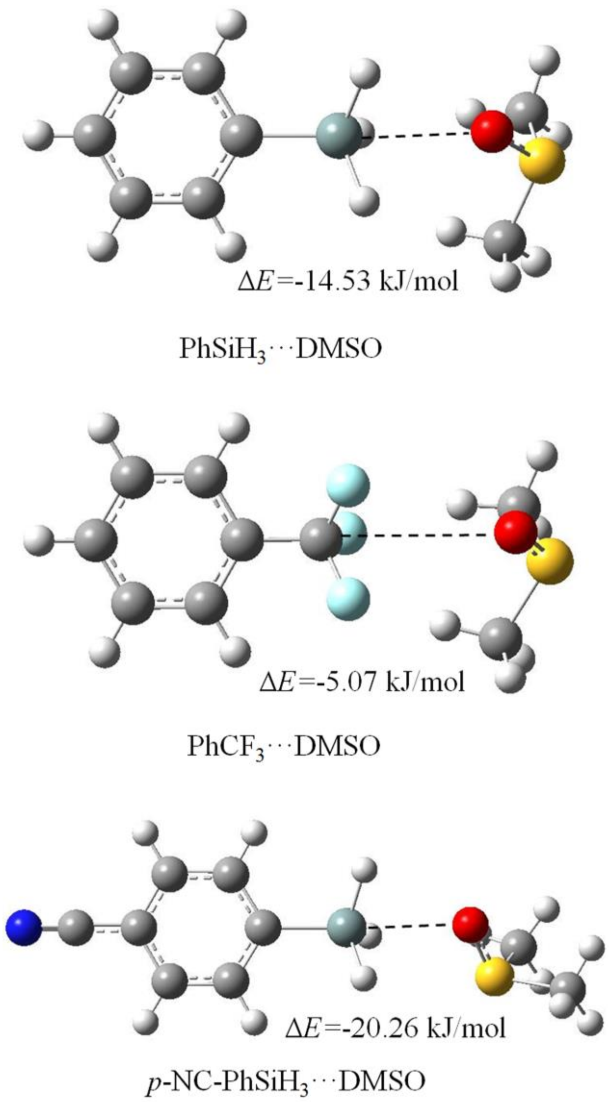

Figure S2 Optimized structures of complexes $PhSiH_3$···DMSO, $PhCF_3$···DMSO and *p*-NC-$PhSiH_3$···DMSO at the MP2/aug'-cc-pVTZ level

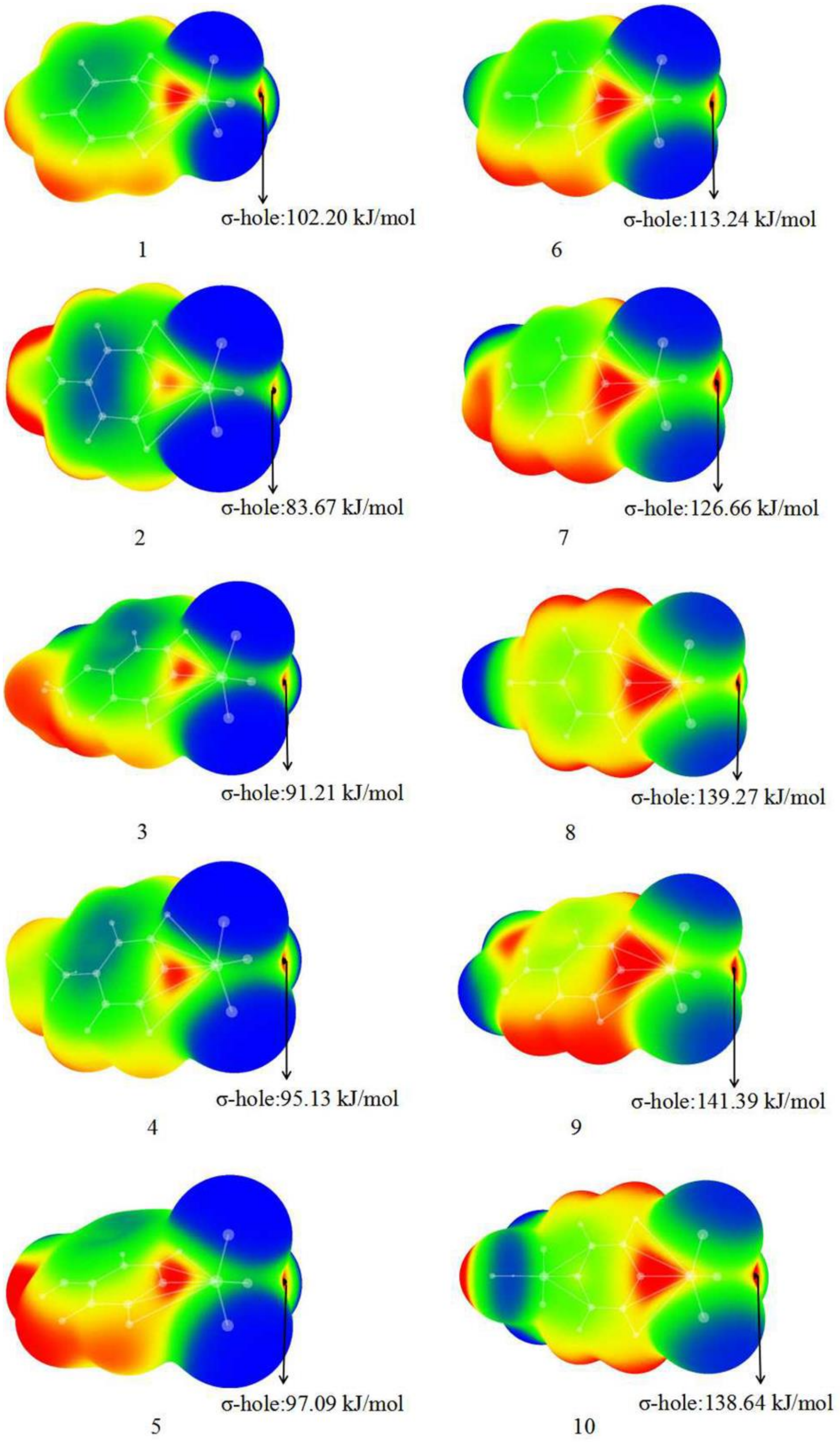

Figure S3 MEP maps of *p*-X-PhSiF_3 (1: X = H, 2: X = NH_2, 3: X = OCH_3, 4: X = CH_3, 5: X = OH, 6: X = F, 7: X = CHO, 8: X = CN, 9: X = NO_2, 10: X = SO_3H)

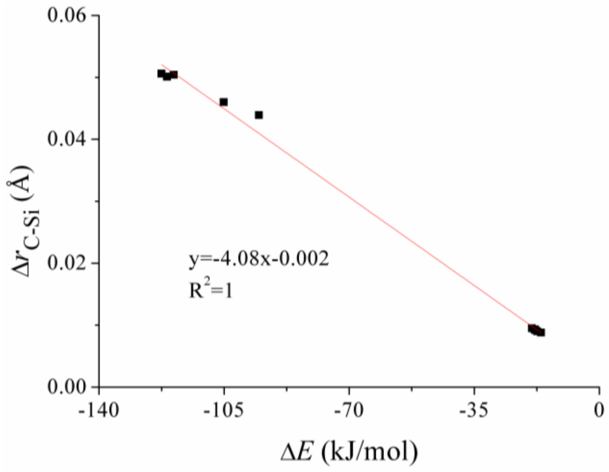

Figure S4 Elongation of C-Si bond ($\Delta r_{\text{C-Si}}$) versus the interaction energy (ΔE) in the complexes *p*-X-PhSiF_3···DMSO

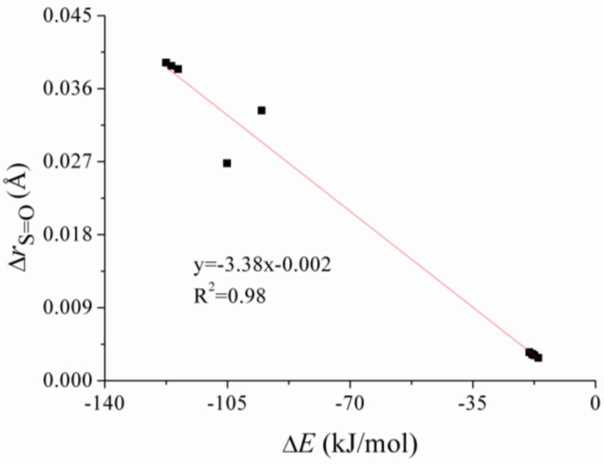

Figure S5 Elongation of S=O bond ($\Delta r_{\text{S=O}}$) versus the interaction energy (ΔE) in the complexes *p*-X-PhSiF_3···DMSO

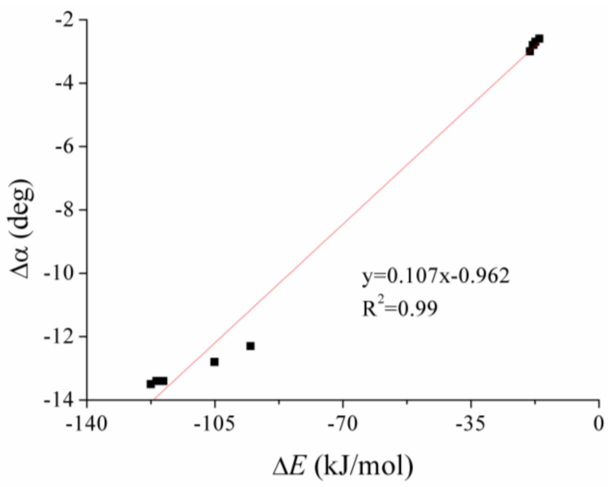

Figure S6 Change of angle C-Si-F (Δα) versus the interaction energy (Δ*E*) in the complexes *p*-X-PhSiF_3···DMSO

Cartesian coordinates of complexes

PhSiF_3···DMSO(1)

C	-2.09873400	-0.03451600	-0.04481600
C	-2.94860800	-1.13054400	0.16587500
C	-2.65119100	1.25530300	-0.04517800
C	-4.31269300	-0.94123400	0.37663300
H	-2.54566900	-2.13627300	0.15682000
C	-4.01494000	1.44465200	0.16550000
H	-2.01719900	2.11684900	-0.21994200
C	-4.84656600	0.34621900	0.37792700
H	-4.95804900	-1.79557100	0.53543400
H	-4.42883100	2.44480500	0.15993300
H	-5.90682700	0.49289100	0.53938400
F	-0.04582200	-1.74994100	-0.82086200
F	0.46348200	-0.07756200	1.11468500
F	0.24701300	0.83929900	-1.27996400
O	2.56572200	-0.76822100	-0.77157700
C	3.18485300	1.69960800	0.02628400
H	2.28950000	1.69896200	0.64418700
H	3.98874400	2.26952100	0.49034100
H	2.96226200	2.09492800	-0.96175800
C	3.82054300	-0.41755700	1.54425400
H	4.56555000	0.21235600	2.02851200
H	2.83151200	-0.27630500	1.97567000
H	4.11562600	-1.46222500	1.60481100
Si	-0.28541500	-0.27992300	-0.28282400

S	3.73721200	-0.00206200	-0.20905300

p-NH_2-$PhSiF_3$···DMSO(2)

C	-1.71577900	-0.10376400	-0.11554300
C	-2.54816900	-1.20938100	0.11396200
C	-2.31230900	1.16513200	-0.16472900
C	-3.91726300	-1.05435600	0.29166100
H	-2.12786700	-2.20771200	0.14516300
C	-3.68086200	1.32456900	0.01226800
H	-1.70693600	2.04405300	-0.35427700
C	-4.50301000	0.21550600	0.24184600
H	-4.54140500	-1.92284800	0.46868100
H	-4.12031100	2.31471400	-0.02895400
F	0.39759700	-1.77271600	-0.83857100
F	0.83669500	-0.09081600	1.09060000
F	0.64101800	0.81646000	-1.30031100
O	3.03661400	-0.74091000	-0.76465200
C	3.54519700	1.73997500	0.06888700
H	2.62449300	1.70769700	0.64733400
H	4.31046400	2.33187300	0.56939700
H	3.35262700	2.13373500	-0.92608100
C	4.18175000	-0.36412100	1.60349300
H	4.88442100	0.28718600	2.12161300
H	3.17086800	-0.25720000	1.99203100
H	4.50782500	-1.39900200	1.67284400
Si	0.09942000	-0.30795300	-0.31236400
S	4.15911000	0.05704500	-0.15068800
N	-5.86608400	0.37900100	0.47866500
H	-6.26703800	1.21600800	0.08441900
H	-6.43023600	-0.43332000	0.28197700

p- OCH_3-$PhSiF_3$···DMSO(3)

C	-2.20706400	-1.11223900	-0.01868800
C	-1.87084500	1.26356800	-0.15777500
C	-3.58212500	-0.92045300	0.12027700
H	-1.82435400	-2.12585200	-0.02534500
C	-3.23461200	1.46902200	-0.02004800
H	-1.22449400	2.12577300	-0.27523700
C	-4.09768600	0.37712100	0.12126300
H	-4.22839100	-1.77989600	0.22167400
H	-3.65788700	2.46506800	-0.02268800
F	0.74105700	-1.81713800	-0.69324400
F	1.19899100	0.03478700	1.08453700

F	1.07245900	0.70972100	-1.38579800
O	3.38701300	-0.86087300	-0.64687500
C	3.96801700	1.67792200	-0.07456100
H	3.04558100	1.73429300	0.49928200
H	4.74993300	2.29562200	0.36532100
H	3.78965800	1.97075800	-1.10629900
C	4.53491900	-0.27198000	1.67590200
H	5.25551700	0.40842500	2.12773300
H	3.52654700	-0.09426900	2.04460100
H	4.82865900	-1.30330000	1.85531200
Si	0.48585100	-0.30205000	-0.30664200
S	4.53120600	-0.03619400	-0.11266000
O	-5.41646800	0.68233700	0.24890600
C	-6.31211600	-0.41247800	0.38918800
H	-7.30105000	0.02515600	0.47678200
H	-6.27561400	-1.06364500	-0.48583100
H	-6.08655200	-0.99128800	1.28649700

p- CH_3-PhSiF_3···DMSO(4)

C	-1.69975400	-0.11216700	-0.11760400
C	-2.53238000	-1.21623700	0.11553600
C	-2.28614400	1.16094200	-0.17203000
C	-3.90340700	-1.04816600	0.29134700
H	-2.11105400	-2.21384400	0.15502900
C	-3.65768900	1.32085300	0.00449900
H	-1.67261300	2.03475500	-0.35948500
C	-4.48693700	0.22025600	0.23749100
H	-4.53015400	-1.91412300	0.47203300
H	-4.09171200	2.31335500	-0.04008200
F	0.41149700	-1.80279300	-0.79000200
F	0.84091900	-0.05795400	1.09565500
F	0.65539100	0.77362300	-1.32940400
O	3.01678200	-0.76212400	-0.72290800
C	3.54421500	1.74567700	0.01357200
H	2.63101600	1.73862400	0.60471000
H	4.31797100	2.35221600	0.48244100
H	3.34049100	2.10369800	-0.99259900
C	4.19157400	-0.30424000	1.61605700
H	4.90368600	0.36171200	2.10160900
H	3.18625400	-0.17815400	2.01319300
H	4.51365700	-1.33758600	1.71875700
Si	0.12123500	-0.32242700	-0.30731500
S	4.14951600	0.05363400	-0.15154100
C	-5.97123400	0.39349000	0.39245300

H	-6.20935400	1.36018800	0.83414900
H	-6.46836400	0.34054200	-0.57768400
H	-6.39335200	-0.38689100	1.02409100

p- OH-PhSiF_3···DMSO(5)

C	-1.71699000	-0.10403500	-0.10549900
C	-2.56294800	-1.20286300	0.09631500
C	-2.29305900	1.17571600	-0.14389300
C	-3.93454600	-1.03184100	0.25921700
H	-2.15447800	-2.20591200	0.11927200
C	-3.66089600	1.35654600	0.01798800
H	-1.67104800	2.04735900	-0.31134800
C	-4.48259300	0.24908300	0.22146100
H	-4.57726000	-1.89203400	0.41116900
H	-4.10713300	2.34164000	-0.01465100
F	0.38079300	-1.83528800	-0.68909400
F	0.82451700	0.00725000	1.10138500
F	0.64223100	0.70865400	-1.36124300
O	2.99084000	-0.81502500	-0.67709800
C	3.53891500	1.72914300	-0.09630800
H	2.62868800	1.76453200	0.49837400
H	4.31922700	2.35732100	0.33139300
H	3.33251000	2.02708800	-1.12132500
C	4.17983800	-0.22443800	1.62454400
H	4.89858600	0.46477600	2.06582900
H	3.17724400	-0.06755400	2.01760600
H	4.49552000	-1.25185900	1.78835600
Si	0.10094500	-0.32955100	-0.28394300
S	4.13180300	0.02600500	-0.16126800
O	-5.81914500	0.48504700	0.37307800
H	-6.27168700	-0.35730800	0.50057200

p- F-PhSiF_3···DMSO(6)

C	1.56763900	0.12367900	-0.08938600
C	2.43240600	1.20617000	0.12620500
C	2.14067100	-1.15213100	-0.21076900
C	3.81282900	1.03293100	0.22248100
H	2.02713300	2.20607100	0.21509400
C	3.51652400	-1.35055200	-0.11803000
H	1.50774300	-2.01372600	-0.38891300
C	4.32598300	-0.24706000	0.09880100
H	4.48352500	1.86559500	0.38608100
H	3.96174000	-2.33164200	-0.21377100

F	-0.61726900	-0.65408500	-1.41432100
F	-0.36608400	1.93058600	-0.34072300
F	-0.70404600	-0.32926900	1.25647800
O	-2.36849400	0.66599300	-0.27116400
C	-3.91222800	-0.25181800	1.65728500
H	-4.30544900	0.76219100	1.69507500
H	-4.68176000	-0.99071900	1.87514400
H	-3.07024000	-0.36428200	2.33507100
C	-4.74907900	-0.15861400	-0.91682100
H	-5.52426600	-0.87988200	-0.66417700
H	-5.04954200	0.85769000	-0.67043300
H	-4.48837900	-0.23524100	-1.96907800
Si	-0.29775400	0.33297300	-0.17393300
S	-3.27070200	-0.54772700	0.01081900
F	5.66281800	-0.42696600	0.18885200

p- CHO-PhSiF_3···DMSO(7)

C	-1.15165800	-0.10242900	-0.21024200
C	-2.03241000	-1.17831100	0.00321100
C	-1.69633400	1.19068000	-0.25166000
C	-3.39686500	-0.97772200	0.17581300
H	-1.64170300	-2.18824500	0.02535900
C	-3.06161100	1.40348000	-0.08186100
H	-1.04909300	2.04131100	-0.42999200
C	-3.91497400	0.31942700	0.13447000
H	-4.07493500	-1.80641800	0.33855100
H	-3.47227400	2.40704900	-0.11978800
F	0.68950800	-1.89951200	-0.85835500
F	1.13389300	-0.03766000	1.16422000
F	1.04141500	0.84827700	-1.38396100
O	2.73284400	-0.74336500	-0.60246600
C	3.49327000	1.76019500	0.07383900
H	2.64348800	1.85385500	0.74472500
H	4.36676300	2.28177100	0.46280200
H	3.24452600	2.12241500	-0.91956900
C	4.10891400	-0.35110800	1.65398400
H	4.95630200	0.21379400	2.04023300
H	3.18455500	-0.09977600	2.16532000
H	4.31127800	-1.41734600	1.71887800
Si	0.70841500	-0.36969200	-0.37166000
S	3.95230200	0.03163200	-0.09338600
C	-5.35860700	0.56585800	0.31231200
O	-6.19391900	-0.30029800	0.50994800
H	-5.65664000	1.63010500	0.25470100

p- CN-PhSiF$_3$···DMSO(8)

C	1.28059700	0.20590100	-0.04888400
C	2.15411800	1.28091800	0.17658500
C	1.85188000	-1.06037000	-0.25490300
C	3.53470300	1.10891900	0.20202200
H	1.75037600	2.27373600	0.32890900
C	3.22841400	-1.25622700	-0.23336200
H	1.21249000	-1.91393800	-0.44563700
C	4.07420600	-0.16541400	-0.00249700
H	4.19647400	1.94778500	0.37516300
H	3.65437600	-2.23795500	-0.39584600
F	-0.62710300	2.01835100	-0.23492900
F	-0.89872100	-0.13848300	1.47409100
F	-0.94180900	-0.69446900	-1.15351200
O	-2.59794500	0.74304100	-0.06837200
C	-3.47140900	-1.63317200	0.78379300
H	-3.49085700	-1.15438600	1.76026800
H	-4.30641400	-2.31908800	0.64801500
H	-2.52841100	-2.15054500	0.63239500
C	-5.14027300	0.35948800	0.03870100
H	-5.93252500	-0.37241500	-0.10768100
H	-5.05116600	0.65567700	1.08141700
H	-5.30635300	1.22819000	-0.59285900
Si	-0.59752900	0.42365200	-0.01423800
S	-3.59092300	-0.36473900	-0.47972100
C	5.49213900	-0.35446000	0.02035700
N	6.65545500	-0.51125600	0.03942500

p- NO$_2$-PhSiF$_3$···DMSO(9)

C	0.89344600	0.23982100	-0.05671000
C	1.75703800	1.33022400	0.12938100
C	1.47642200	-1.02726900	-0.22056300
C	3.14110000	1.17610600	0.15760000
H	1.34405600	2.32357500	0.24885700
C	2.85559300	-1.21343500	-0.19650000
H	0.84484700	-1.89284200	-0.37994800
C	3.66347600	-0.09981500	-0.00552100
H	3.80773000	2.01425700	0.29889900
H	3.30568800	-2.18704300	-0.32413600
F	-1.03104800	2.02721900	-0.29017700
F	-1.27852600	-0.08602000	1.47834800
F	-1.31887000	-0.71382500	-1.13358600

O	-2.98664200	0.73589000	-0.08532700
C	-3.83309100	-1.62973900	0.82210500
H	-3.85540100	-1.12953500	1.78770900
H	-4.66110200	-2.32727100	0.70375800
H	-2.88508600	-2.14041300	0.67954300
C	-5.52406900	0.32907100	0.03715300
H	-6.30935900	-0.41373300	-0.09065300
H	-5.43527300	0.64943000	1.07271600
H	-5.70030900	1.18167000	-0.61332000
Si	-0.98727300	0.43935300	-0.02544300
S	-3.96916100	-0.39146100	-0.46915500
N	5.12040100	-0.27871900	0.02154000
O	5.81622600	0.72245000	0.19004900
O	5.55870500	-1.41968700	-0.12528000

p- SO_3H -$PhSiF_3$···DMSO(10)

C	-0.24158900	0.27822800	0.01668900
C	-1.08264200	1.37258800	-0.23654400
C	-0.84383100	-0.97529900	0.21624600
C	-2.46851200	1.23455000	-0.28878100
H	-0.65077100	2.35248600	-0.39577500
C	-2.22471300	-1.14319800	0.16820200
H	-0.22587200	-1.84199900	0.41777000
C	-3.01940700	-0.02670200	-0.08057800
H	-3.11366900	2.07722400	-0.50004900
H	-2.68345800	-2.11270900	0.31125100
F	1.70215300	2.04415500	0.25515400
F	1.94555800	-0.09588900	-1.48179100
F	1.94144900	-0.68654200	1.14225700
O	3.64192800	0.72291400	0.09401500
C	4.46335300	-1.66381900	-0.78304600
H	4.50177600	-1.17324800	-1.75305200
H	5.28090100	-2.37093700	-0.64970000
H	3.50738800	-2.16082200	-0.64536000
C	6.17399500	0.27825000	0.00108700
H	6.94725100	-0.47431300	0.14421900
H	6.10040800	0.58963300	-1.03839300
H	6.35559300	1.13455400	0.64517400
Si	1.64293500	0.45329300	0.01116600
S	4.60399900	-0.41551800	0.49801500
S	-4.77282600	-0.21948400	-0.11766700
O	-5.36601300	0.94666300	-0.74126500
O	-5.09244000	-1.56295900	-0.52912200
O	-5.15809500	-0.16309600	1.45649000

H	-5.36481200	0.76277500	1.66084800

p-SiF_3-PhOH···DMSO(11)

C	-1.90133900	0.27664100	-0.12385600
C	-0.97810100	-0.46376600	-0.87740700
C	-1.46655400	1.47097800	0.47339000
C	0.33714100	-0.03635300	-1.02468300
H	-1.28576700	-1.37985600	-1.36851000
C	-0.15444000	1.90403100	0.33349000
H	-2.15805700	2.07537600	1.04881500
C	0.75708900	1.15011000	-0.41123700
H	1.04331800	-0.60639300	-1.61608000
H	0.18227600	2.82612300	0.78946600
F	-4.10283700	-1.17929200	-1.14233000
F	-4.62973400	0.89039100	0.30701800
F	-3.78586300	-1.25727200	1.38135000
O	3.59340800	-0.39715000	-1.25486400
C	4.94277000	0.50128500	0.84936300
H	4.16367200	1.25637900	0.94411300
H	5.32222800	0.19102200	1.82178600
H	5.75674400	0.86497000	0.22717300
C	2.91644900	-1.23532800	1.18498300
H	3.35540900	-1.54730500	2.13183700
H	2.32539200	-0.32823100	1.29961500
H	2.30738600	-2.03646500	0.77279700
Si	-3.61419900	-0.31871600	0.10471200
S	4.24884500	-0.93251500	0.01190600
O	2.02700500	1.60554600	-0.50841000
H	2.58918000	0.90944800	-0.93989900

p-SiF_3-$PhNH_2$···DMSO(12)

C	-1.44679100	0.54287000	0.11179200
C	-1.19058000	1.48461000	-0.89749200
C	-0.50885000	0.41605200	1.15028200
C	-0.02565700	2.24274600	-0.88897000
H	-1.90139700	1.62529900	-1.70351600
C	0.65130000	1.17793500	1.16405100
H	-0.68343400	-0.28065300	1.96263800
C	0.92757000	2.08213500	0.12611500
H	0.16179600	2.95171600	-1.68757800
H	1.36380000	1.07717800	1.97316700
F	-4.05922300	0.09646300	-0.87340200
F	-3.53735300	-0.78886700	1.49388400

F	-2.60524400	-1.97453500	-0.55019500
O	3.54035800	0.21675200	0.30325300
C	1.77989500	-1.06429600	-1.23925000
H	2.45446800	-0.83090500	-2.06242600
H	1.31753300	-2.04265000	-1.36751000
H	1.01287400	-0.29869600	-1.13875400
C	3.90114300	-2.34900000	-0.21712700
H	3.36548500	-3.28513100	-0.36744600
H	4.39783400	-2.02187800	-1.12914900
H	4.62293900	-2.45248800	0.58917000
Si	-2.92309300	-0.53011500	0.04861900
S	2.73813000	-1.06799400	0.28599600
N	2.10834600	2.80515400	0.14763800
H	2.31755400	3.26941000	-0.72405500
H	2.89068800	2.23237700	0.45037900

p-SiF_3-PhCHO···DMSO(13)

C	-0.41137300	-1.07388300	1.41010700
C	0.88044500	-0.56812800	1.31457300
C	1.53592100	-0.52297700	0.07263300
C	0.87796200	-1.01973600	-1.06654700
C	-0.41195100	-1.52985000	-0.96869000
C	-1.06173500	-1.54251300	0.26779900
H	-0.93486300	-1.07823500	2.35840900
H	1.37786300	-0.20278900	2.20557600
H	1.37896200	-1.01284800	-2.02760600
H	-0.93253100	-1.91779100	-1.83613400
C	-2.45988000	-2.03206900	0.37019800
H	-2.86796300	-2.08828700	1.39039500
O	-3.12204000	-2.36069400	-0.59915200
O	-2.98045200	0.54999900	1.20172100
C	-1.25018700	2.06646200	-0.14983100
H	-0.75375300	1.15311000	-0.47528600
H	-1.21276900	2.83401500	-0.92230700
H	-0.79962000	2.43839400	0.76766100
C	-3.38451400	1.00652200	-1.38640100
H	-3.30629400	1.78902200	-2.14016500
H	-2.70857600	0.17954100	-1.59643200
H	-4.40744200	0.64288400	-1.32307800
S	-2.97913900	1.70480800	0.22573800
F	3.99671900	-0.36853200	-1.31401200
F	3.14176900	1.80082200	-0.27228600
F	4.06668800	-0.02841800	1.24540800
Si	3.20846000	0.22384200	-0.06774500

p-SiF_3-$PhNO_2$···DMSO(14)

C	-2.07671000	0.22371100	0.02416400
C	-1.44192500	0.43542100	1.25793900
C	-1.37410900	0.50236100	-1.15962400
C	-0.13717700	0.91330600	1.31523300
H	-1.96222600	0.21879000	2.18306000
C	-0.07366100	0.98971800	-1.12095700
H	-1.83780000	0.32418800	-2.12214600
C	0.51610900	1.18226400	0.12039000
H	0.37145800	1.07379300	2.25452300
H	0.49194600	1.18912500	-2.01847400
F	-4.12688000	-1.30992100	1.21772100
F	-4.06780600	-1.22588400	-1.36376200
F	-4.87365200	0.79045500	-0.02283900
O	2.16563500	-0.94842800	-0.73468600
C	4.73215800	-0.25535500	-0.63521300
H	4.43029000	0.61044800	-0.04796600
H	5.70749700	-0.62859700	-0.32520200
H	4.74416700	-0.00821000	-1.69411400
C	3.61702900	-1.64131900	1.37498000
H	4.61914400	-1.95460400	1.66473200
H	3.37428900	-0.65668100	1.77221400
H	2.88502400	-2.37696400	1.69967600
Si	-3.80801600	-0.38926100	-0.03827500
S	3.51221900	-1.56525500	-0.42335000
N	1.88744900	1.68514700	0.17032000
O	2.49346400	1.58689600	1.23984100
O	2.35659300	2.17577100	-0.85398500
